# From Bench-Scale Experiments to Technical Scale-Up: Rethinking Sorption Research for the Removal of Trace Organic Contaminants from Water

**DOI:** 10.1021/acs.est.6c07585

**Published:** 2026-10-23

**Authors:** Damian E. Helbling, Detlef R. U. Knappe, Joseph J. Pignatello, J. Conrad Pritchard, Wenqing Xu

**Affiliations:** † School of Civil and Environmental Engineering, 5922 Cornell University, 220 Hollister Hall, Ithaca, New York 14853-3501, United States; ‡ Department of Civil, Construction, and Environmental Engineering, North Carolina State University, 915 Partners Way, Raleigh, North Carolina 27695-7908, United States; § Department of Environmental Science and Forestry, 5758 The Connecticut Agricultural Experiment Station, 123 Huntington Street, New Haven, Connecticut 06511, United States; ∥ O’Neill School of Public and Environmental Affairs, Indiana University, 1315 E. Tenth Street, Bloomington, Indiana 47405-1701, United States; ⊥ Department of Civil and Environmental Engineering, 8210 Villanova University, 800 E. Lancaster Ave., Villanova, Pennsylvania 19085, United States

**Keywords:** trace organic contaminants, water treatment, remediation, mass transfer, sorption kinetics, sorption isotherm, novel sorbents, scale-up

## Abstract

Drinking water, wastewater, and stormwater systems are increasingly challenged by trace organic contaminants (TrOCs), including PFAS, pharmaceuticals, pesticides, and other persistent and highly mobile contaminants that occur at ultralow concentrations in complex water matrices. Sorption-based treatment processes remain central to TrOC removal because they can concentrate dilute contaminants without generating potentially toxic transformation products. However, much of the current sorption literature remains disconnected from the needs of performance prediction and technical scale-up. Equilibrium and kinetic parameters are often derived under simplified laboratory conditions, using empirically fitted models, elevated contaminant concentrations, and/or nonrepresentative water matrices, limiting their physical interpretability and transferability across treatment scenarios. This review critically examines three interconnected areas governing the engineering relevance of sorption data: thermodynamic principles governing sorption equilibria, mechanistic interpretation of sorption kinetics, and the evaluation and transition of novel sorbents to scaled treatment systems. By identifying limitations in prevailing experimental practices and aligning equilibrium characterization, kinetic modeling, and sorbent evaluation with application-relevant conditions, this review provides a framework for generating mechanistically grounded sorption data to support material selection and translate laboratory-derived sorption data into process design parameters for pilot- and full-scale TrOC treatment systems.

## Graphical abstract

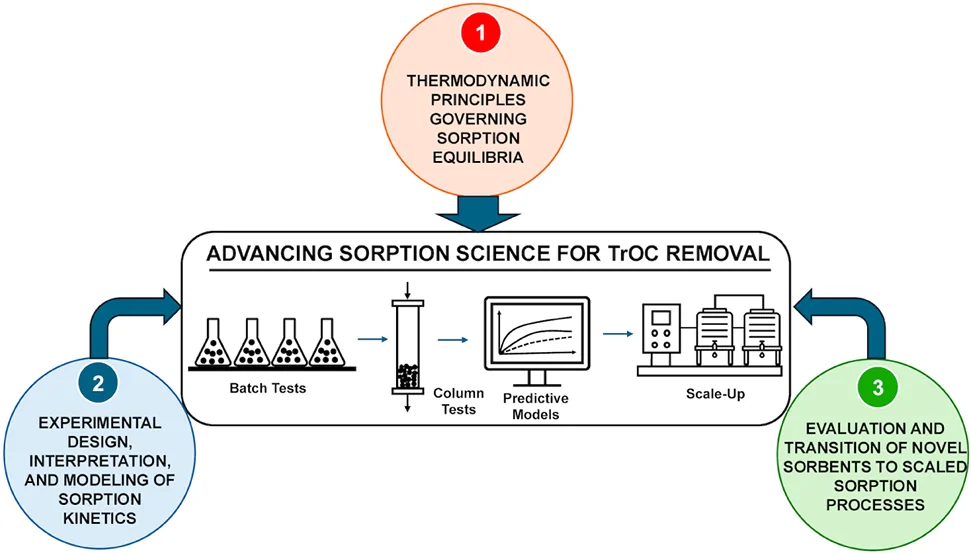

## Scope and Motivation

Trace organic contaminants (TrOCs) encompass a broad range of chemicals encountered in drinking water, wastewater, and stormwater,^1,2^ including legacy hydrophobic compounds, per- and polyfluoroalkyl substances (PFAS), pesticides, pharmaceuticals, personal care products, and tire-derived organic chemicals, among others. Many TrOCs raise toxicological concerns because of their persistence, bioaccumulation potential, mobility, biological activity, and ongoing environmental releases, particularly in complex mixtures.^3–6^ Treatment technologies such as biodegradation and advanced oxidation processes are ill-suited for many TrOCs because of slow kinetics and incomplete mineralization. A given water may contain dozens of TrOCs with diverse physicochemical properties, including charge, molecular size, hydrophobicity, biological reactivity, and chemical reactivity toward a given treatment strategy.^7,8^ Even TrOCs within the same use class can differ substantially in these properties. For example, triazine herbicides are structurally similar but differ in charge state, molecular volume, and hydrophobicity at circumneutral pH, which can strongly influence their removal in treatment processes. Compounding this challenge, individual TrOCs may be present at very low concentrations, ranging from ∼ 100 μg L^–1^ down to analytical detection limits, while co-occurring matrix constituents, such as dissolved organic matter (DOM) and inorganic ions, are present at concentrations several orders of magnitude higher.^9^ Under these “needle-in-a-haystack” scenarios, removal technologies that are selective for the needle (i.e., target TrOCs), while minimizing interferences from the haystack (i.e., DOM and other matrix constituents), are often desired.

Sorption-based treatment processes remain central to TrOC removal from water because they can cost-effectively remove and concentrate otherwise dilute TrOCs, enabling more efficient downstream processes that mineralize the sorbed TrOCs. Some carbonaceous sorbents, such as activated carbon (AC), are widely used in drinking water and wastewater treatment,^10–13^ while other sorbents, such as biochar and surface-modified biochar, engineered polymers, ion-exchange resins, covalent-organic frameworks (COFs), and metal–organic frameworks (MOFs), are being developed with increasing emphasis on sorbate selectivity through tailored surface chemistry.^14–18^ Sorption processes capture TrOCs without transforming them into oxidation or disinfection byproducts, which may be equally or more toxic than the parent compounds.^19,20^ Sorption processes also remove TrOCs from a waste stream to very low levels over short times, compared to direct chemical or biological treatment, which often require longer times and/or high reagent concentrations to treat TrOCs. Beyond conventional drinking water and wastewater treatment, sorbent-based materials are increasingly used in stormwater management systems to sequester TrOCs in runoff before transport to receiving waters.^21^ In these systems, sorbents are either deployed in *ex situ* treatment configurations, such as modular treatment units, or incorporated into *in situ* green infrastructure as amendments.^22–24^

Despite these advantages and the widespread use of sorption-based treatment, the current body of sorption research for TrOCs remains largely disconnected from the needs of performance prediction and technical scale-up. Many laboratory studies determine empirically fitted equilibrium and kinetic parameters that lack clear physical meaning and are inherently tied to specific experimental conditions. For example, sorption capacities are typically obtained under non-equilibrium conditions, at elevated TrOC concentrations, or in simplified water matrices that do not capture competition from water matrix constituents. Similarly, kinetic studies often rely on empirically fitted rate constants that cannot be readily compared across systems, linked to underlying transport processes, or used for predictive design. Differences in sorbent particle size and material form between laboratory testing and intended applications can further limit the transferability of sorption data. Promising sorbents are often evaluated without long-term or process-relevant validation and thus fail to capture the complexity of engineered treatment systems. Collectively, these limitations reduce the comparability, interpretability, and predictive value of sorption data needed to guide material selection, process design, and technical scale-up for real-world applications.

This review critically examines three interconnected areas where prevailing practices limit mechanistic understanding and engineering applicability: the thermodynamic principles governing sorption equilibria; the experimental design, interpretation, and modeling of sorption kinetics; and the evaluation and transition of novel sorbents to scaled sorption processes. For each area, we identify gaps in current practice, review the mechanistic and modeling frameworks needed to address them, and outline experimental approaches that yield data with greater predictive value. We also discuss the role of emerging data-driven and machine-learning methods in accelerating sorbent discovery and design. The overarching aim is to advance sorption science that is more mechanistically grounded, rigorous in both design and interpretation, and applicable to the engineering challenges of TrOC removal.

## Thermodynamic Principles Governing Sorption Equilibria

Sorbents under study or development are typically highly porous with large surface areas, and many, especially carbonaceous sorbents (e.g., AC, biochar), are structurally anisotropic, whereas others derive selectivity primarily from ion exchange. Interpreting thermodynamic data on their interactions with TrOCs is therefore complex. However, a clear understanding of the underlying physicochemical principles is essential for generating data that are mechanistically meaningful and transferable across systems. The following sections review these principles as a foundation for identifying where current practices frequently fall short.

### Thermodynamic Constants

Establishing sorption isotherms under realistic conditions is essential not only for understanding mechanisms but also for comparing different sorbents, comparing different TrOCs on a single sorbent, evaluating the effects of potential competing solutes, and applying mass transfer laws that require the input of sorption equilibrium parameters and are essential for predicting sorbent performance in scaled treatment processes. Many publications report removal efficiencies at a given contaminant dose. But such values are meaningless without information on the solid-to-liquid ratio, the presence and concentrations of competing substances, and whether the employed concentration is relevant to the remediation effort.

The most widely used equilibrium sorption isotherm models are the dual-parameter *Freundlich* and *Langmuir,* and the three-parameter *Dubinin-Ashtakhov* (DA, also referred to as Polanyi-Dubinin-Manes) and *Dual-Mode* models (Table 1). Variations on these models exist but require additional fitting parameters. When comparing goodness-of-fit among models, the *adjusted* coefficient of determination (*r*_adj_^2^) is required because it accounts for the number of fitting parameters (degrees of freedom). The *Linear* model (Freundlich *N* = 1) rarely holds for highly porous, tailored sorbents desirable for selective removal of TrOCs from water. Nonlinearity is a “fact of life” for these sorbents and has major consequences for solute transport through sorbent columns: self-sharpening fronts and concentration-dependent breakthrough.

**Table 1:** Common Sorption Isotherm Models Relating Sorbed Concentration (*q*) to Solution Concentration (*C*_w_)

Model	Equation	Definitions
Freundlich	$q=K_{F}\cdot {C_{w}}^{N}$	*K*_F_, capacity-affinity parameter; *N*, linearity exponent (0–1).
Langmuir	$q=\frac{Q_{L}K_{L}\cdot C_{w}}{1+K_{L}C_{w}}$	*Q*_L_, maximum capacity; *K*_L_, affinity coefficient.
Dubinin-Ashtakhov	$$\begin{array}{l} \ln\frac{q}{q^{\max}}=-{\left(\varepsilon /E_{\mathrm{DA}}\right)}^{b}\\ \varepsilon =-RT\ln a_{w} \end{array}$$	*q* ^**max**^, maximum capacity; *E*_DA_(kJ mol^–1^), characteristic adsorption energy relative to a reference compound; *b,* site heterogeneity parameter (typically, 1 – 3); *a*_w_, solute activity in water.
Dual Mode	$$q=K_{p}C_{w}+\frac{q_{h}^{\max}K_{h}C_{w}}{1+K_{h}C_{w}}$$	*K*_p_, partition domain sorption coefficient; *K*_h_ and $q_{h}^{\max}$ , “hole-filling” domain affinity and capacity coefficients.

Nevertheless, sorption can in general be taken as approximately linear over a narrow concentration range (while ignoring the regression intercept), and all sorption is linear in the limit of infinite dilution. This is particularly relevant for TrOCs which are usually at very low concentrations.

The Langmuir model is a general saturation model that assumes a limited population of uniform energy sites, which is rarely realistic for carbonaceous and other structurally heterogeneous sorbents. The predicted linearity at the lower-concentration end of the Langmuir is usually belied by the curvature of the data observed there. The Freundlich model is a general power-law model but can be derived from the Langmuir model by assuming a distribution of site energies over an infinite range. The unlimited energy as *C*_w_ approaches zero makes the Freundlich model unrealistic at very low concentrations. Some experimental evidence is illustrative: Braida constructed a detailed isotherm of benzene in a char covering 5 orders of magnitude in aqueous concentration, ranging from the method quantification limit to about half the water solubility.^25^ The “running Freundlich fit” approached linearity at both the very low- and the very high-concentration ends. Thus, nonlinearity is most pronounced in the (log scale) middle concentration range. The main value of the DA model is that it yields sorption energy (kJ mol^–1^) relative to a reference compound. The *E*_DA_ represents the sum of all forces of direct interaction between the molecule and the solid.^26,27^ Comparison of *E*_DA_ among different compounds for a given sorbent, or for a single compound among multiple sorbents, is strictly valid only when sorbed concentration (*q*) is 10% of the maximum capacity (*q*_max_), at which the site heterogeneity fitting parameter (*b*) in the DA model is irrelevant. For structurally similar TrOCs with different functionalities, DA-derived energy parameters may provide useful mechanistic insight into how surface properties may influence selectivity. The Dual-Mode model is commonly used for polymers that present a “partition” domain (linear term) and a “hole-filling” domain (Langmuir-type term) to the sorbate.

The fit of a particular model can be used to support, but never to assign a specific sorption mechanism. Nevertheless, all models are useful for pinpointing a partition coefficient *K*_d_ (*K*_d_=*q*/*C*_w_, where *C*_w_ is the aqueous concentration at equilibrium) along the isotherm; for example, for the Freundlich model, $K_{d}=K_{F}\cdot C_{w}^{N-1}$ . The magnitude of *K*_d_ is useful for comparing different sorbates and for evaluating new sorbents against a benchmark. Comparison of *K*_d_ values for a single sorbate on multiple sorbents must be made at the same *C*_w_. Likewise, comparison of different sorbates *i* on a single sorbent must be made at the same *chemical activity* of *i*, defined as the fractional saturated water-solubility, $C_{w,i}/C_{w,i}^{\mathrm{sat}}$ . Comparisons are most advantageously made at TrOC-relevant (i.e., low) concentrations. Sorption is often temperature-dependent, which has implications for the transferability of lab data to real-world situations. Sorption of neutral compounds by carbonaceous sorbents is typically exothermic, while the temperature-dependence of sorption by ion exchange resins varies. The enthalpy of sorption (Δ*H*_*sorp*_) (equal to the negative of the isosteric heat of sorption) can be obtained from the temperature dependence of *K*_d_, under the assumption that physical changes in the sorbent are negligible over the experimental temperature range. This assumption is probably valid for carbonaceous sorbents, which are made at high temperatures, but polymers and MOFs may be more sensitive to temperature changes. Note that enthalpy applies to a specific *fractional coverage*, *q*/*q*_max_,^28^ where *q*_max_ may be taken at the isotherm plateau or at the value of *q* extrapolated to the water solubility. Comparisons of Δ*H*_*sorp*_ for a single compound on different sorbents, or for multiple compounds on a single sorbent, should be made at the same *q*/*q*_max_. This is a case where the full isotherm must be constructed. The related Gibbs parameters, free energy (Δ*G*_sorp_) and entropy of sorption (Δ*S*_sorp_) are, strictly speaking, unobtainable because the standard sorbed state cannot be defined.^28^ Apparent values reported in the literature should be interpreted with caution. Even if obtainable, the Δ*G*_sorp_ would be no more useful than the *K*_d_. Moreover, knowing Δ*S*_sorp_ is of limited value without knowing the changes in the waters of hydration of both the sorbent and the sorbate upon sorption, which are generally unknown.

### Competitive Sorption

TrOCs are often present in mixtures, yet competitive effects are frequently ignored. This omission can lead to overestimation of sorption performance under realistic treatment conditions. In addition to other TrOCs, DOM may serve as a competing solute, and ionic/ionizable TrOCs may be subject to competition from inorganic ions present in the water.^29,30^ Competitive solutes in the water being treated may vary in concentration and/or composition over time. Competitive sorption can be expected for any sorbent with a finite number of sorption sites or a heterogeneous distribution of sorption site energies. Such properties are characteristic of most sorbents regarded as candidates for water treatment. The strength of the competitive effect depends on the concentrations of the competing solute(s) and the degree of overlap in their inherent sorption domains. Competitive effects have been observed at environmentally relevant TrOC concentrations, for example, PFAS on clay, AC, and modified biochars.^17,31–35^ Competitive effects have been observed between species of different polarity and even charge (e.g., between TrOCs and DOM), highlighting that competition is governed not simply by chemical similarity, but by overlap in sorption domains.^36,37^ Besides competing for specific sorption sites, DOM can block entrances to small pores, reducing the accessible sorption domain for TrOCs.^29,30,38^ Classical experiments on AC show that preadsorption of vapors of long-chain alkyl hydrocarbons, representatives of some moieties in DOM, can block pore entrances and suppress sorption of a second solute.^39^ At a given concentration of a competing solute, the competitive effect increases with decreasing equilibrium concentration of the TrOC.^40^ Apart from reducing the magnitude of TrOC sorption, competition can increase the desorption rate (by occupying some of the higher-energy, lower mobility sites), increase the apparent linearity of the isotherm (by competing more effectively at the lower-concentration sites),^41^ increase the degree of hysteresis (if the competing solute is also diluted in the desorption step),^42^ and increase bioavailability (by displacing more TrOC into solution).^43^

DOM composition can vary greatly among drinking water, wastewater, and stormwater matrices, and thus the source and characteristics of DOM can affect the extent of competition and pore blockage.^44^ Generally, lower-molecular-weight DOM (<500 Da) sorbs more readily to AC and directly competes with TrOCs for sorption sites.^45–48^ In contrast, higher-molecular-weight DOM is less sorbable by activated carbon because of size-exclusion and is more likely to interfere with TrOC sorption through pore blockage.^49^ Sorption competition is effectively modeled with Ideal Adsorbed Solution Theory (IAST).^50^ The IAST assumes: *i*) the sorbent presents the same surface to all compounds, albeit with different affinities; and *ii*) under dilute conditions, the sorbate mixture forms a two-dimensional ideal solution on the surface, such that the fugacity of each sorbate is proportional to its mole fraction times its fugacity in the single-compound system at the same spreading pressure. IAST is adaptable to any sorption model, in principle, but solutions are available for the Freundlich model. The application of IAST requires the full single-solute isotherm for each competing solute in the system. Partial noncompliance with IAST for a competing pair has been interpreted as incomplete sharing of sorption domains.^51^ The IAST has also been successfully applied to describe adsorption equilibria of TrOCs in the presence of DOM by representing the DOM by one or more fictive components.^52,53^ In principle, all the competitive substances, including DOM and other TrOCs, can be represented as one hypothetical “equivalent background compound”.^52^ In ion-exchange sorbents, equilibrium descriptions often explicitly account for competing ions and the exchange stoichiometry.^54^

### Structural Effects

The design of new sorbents or the modification of conventional sorbents can benefit from current understanding of the nature of high-surface-area porous carbonaceous sorbents for TrOC removal. For such sorbents, the vast majority of surface area—hence, sorption sites—lies in the interior micropore and mesopore networks. Thus, nominal particle size has little effect on *K*_d_ of small-to-medium size molecules that can enter and interact with those small pores and that are hardly affected by external surface area. However, particle size has a much greater effect on the *K*_d_ of large molecules, such as polymers (e.g., polystyrenesulfonates) and DOM, which are more restricted to external and larger-pore surfaces.^55^

Sorption of TrOCs generally increases with the specific surface area of the sorbent, but the relationship is often nonlinear, and the rule may break down when pore accessibility or surface chemistry are more important. The reasons are not fully understood, but the following considerations offer some guidance. First, common surface area measurement techniques are not ideal for determining the surface area accessible to organic solutes in water. Specific surface area (SSA, m^2^ g^–1^) is customarily determined by gas porosimetry using small probe gases (N_2_, CO_2_). These gases can probe pores as small as 0.4 nm wide, whereas most small organic molecules can access pores down to ∼ 0.7 nm.^12^ Gas adsorption is typically measured after outgassing at elevated temperature, which removes water (and volatile organics) confined within pores that, in solution, would compete with the solute. The choice of probe gas, despite their close similarity in molecular size, can strongly affect the SSA value. Nitrogen up to 1 bar at 77 K can probe both micropores (<2 nm wide) and mesopores (2–50 nm), while CO_2_ up to 1 bar at 273 K can probe only micropores. Yet, the SSA of microporous or mixed microporous/mesoporous carbonaceous sorbents is typically much greater when determined by the CO_2_ than the N_2_ method. The discrepancy arises because N_2_ diffusion in micropores is slow at 77 K, so micropores are poorly accessed.^25,39,56^ A second cause for the lack of correspondence between sorption and SSA is pore surface chemistry. For example, oxidation of carbonaceous sorbents by hot air or chemical reagents, despite having only a minor effect on SSA (even slightly increasing it), usually leads to a strong decline in sorption because water molecules “crowd out” contaminant molecules by clustering around polar sites.^12,57,58^ A third reason is pore size distribution. Incoming molecules not only occupy “sites” on pore walls but also condense into liquid-like or solid-like states that fill up pore spaces and displace some of the water initially present there. “Pore filling”, as it is known, is the basis for popular sorption models, such as the DA and others based on Polanyi theory. The filling of small pores helps explain the remarkable adsorption capacity of carbonaceous sorbents for many TrOCs at environmentally relevant concentrations. Nanoconfinement in small pores can affect the physical states of the sorbate and water. Organic compounds that are liquids in their standard state at ambient temperature may freeze inside a carbon nanopore due to stronger interactions with the hydrophobic pore wall than with one another;^59^ as examples, in the micropores of AC fibers, the freezing point of CCl_4_ rose from −22.9 to 34.1 °C, benzene from 5.5 to 65.5 °C, and aniline from −6 to 25 °C. Freezing-point elevation in small pores could affect hysteresis and desorption kinetics. The opposite can occur (freezing-point depression) with a hydrophobic compound in hydrophilic pores, or with water in hydrophobic pores. For example, in AC micropores, water stays liquid-like at temperatures above −36 °C.^60–62^ Nanoconfinement in carbonaceous pores can affect the density, orientation, and permittivity of water layers,^63^ as well as the concentration, spatial distribution, and orientation of H_3_O^+^, OH^–^, and common inorganic cations and anions.^64–68^

Pore accessibility is governed by sorbate steric size and shape in relation to pore size. Molecules of different sizes access different subsets of pores. Molecules are excluded (*molecular sieving effect*) once their critical molecular dimension (CMD) reaches ∼ 90% of the critical pore dimension, which is the diameter of the smallest cylindrical pore into which the molecule can fit. Most TrOCs have effective molecular diameters between 0.5 and 2 nm. For reference, the van der Waals diameter of CCl_4_ is 1.12 nm. Perfluoroalkanoic acids of carbon chain lengths 4 to 9 have effective cross-sectional diameters of 0.45–0.72 nm and maximum diameters of 0.96–1.54 nm.^69^ Thus, ordinary compounds can access pores down to the supermicropore size (0.7–2.0 nm). Li et al. estimated that dilute TCE or methyl-*t*-butyl ether (at ≤ 4 × 10^–5^ times water solubility) mainly occupied AC pores of, respectively, 0.7–1.0 or 0.8–1.1 nm, corresponding to 1.3–1.8 times their kinetic diameters.^12^ Steric hindrance in microporous materials begins to reduce molecular diffusivity when the CMD reaches ∼ 10% of the pore width and becomes severe at ∼ 90%.^39^

### Practical Issues

Prediction of TrOC removal during scale-up to packed-bed adsorbers requires reliable equilibrium relationships that describe sorption of target TrOCs under relevant water chemistries, including competition from background matrix constituents, because these relationships are key inputs to models of packed-bed performance and breakthrough. While structural characterization provides important insight into pore accessibility and sorption domains, interpreting these relationships assumes sorption equilibrium is thermodynamically well-defined. However, sorption by carbonaceous sorbents is not necessarily well-behaved thermodynamically. Isotherms often display hysteresis, which is manifested by the displacement of the desorption branch above the sorption branch of the isotherm. True hysteresis—as opposed to artificial hysteresis resulting from mass loss or insufficient equilibrium time—is due to the formation of metastable state(s).^70^ Capillary condensation hysteresis manifests in fixed-pore solids. A contaminant entering cylindrical or slit mesopores or ink-bottle-shaped pores sorbs to a different (metastable) state than from which it leaves.^39^ The true thermodynamic isotherm of solids containing such pores is considered to be the desorption branch. Pore deformation hysteresis applies to more flexible solids such as glassy polymers^41,42,70^ and hard organic matter.^41,42,70^ It has also been invoked for biochars^25^ and ACs.^71^ The incoming sorbate exerts local pressure on internal pores or proto-pores, causing inelastic pore expansion and pore creation. Desorption (or second-time sorption) from the now-expanded, now-more-microporous material follows a displaced isotherm. Neither branch is the true thermodynamic isotherm, since the sorbent has changed structurally during the process. Which branch should be used in modeling for purposes of TrOC remediation is a valid question. No study to our knowledge has addressed this question explicitly in the context of miscible displacement. Batch experiments on a brown coal using tiny amounts of ^14^C-tracers showed free exchange between sorbed and dissolved molecules at specific places along the (hysteretic) sorption or desorption branches of the isotherm, confirming the observed hysteresis is due to sorbate-induced physical changes in the sorbent, and therefore “equilibrium” depends on whether uptake or release is being probed.^41^ In miscible displacement, a solute experiences diffusion both in and out of sorbent particles while progressing along a column. If equilibrium is assumed to be reached, the front of the eluting peak at any given point along the column reflects predominantly diffusion into particles (net sorption), while the tail of the peak reflects predominantly net desorption. If equilibrium is not reached, the balance shifts toward net sorption. Equilibrium is usually not reached in granular activated carbon (GAC) applications, so for practical column design with these materials, the sorption branch may better reflect the equilibrium state up to breakthrough. The discrepancy will depend, of course, on the degree of hysteresis. After breakthrough (chromatographic peaking), the desorption branch may better reflect true equilibrium.

Careful experimental design is required to minimize artifacts and generate isotherm parameters that remain useful for mechanistic interpretation and predictive modeling. Major considerations include the range of TrOC concentrations, equilibration time, solids concentration, and background solution chemistry. Equilibrium aqueous TrOC concentrations should ideally span the full scale, ranging from a few times above the method detection limit to roughly half solubility, or when sorption reaches saturation. Although TrOCs are highly dilute by definition, saturation values are needed to apply the Langmuir, DA, and Dual-Mode models and to compute ΔH_sorp_. Data collection should favor the lower end of the isotherm, where field TrOC concentrations are expected to range. Weighted least-squares regression should be used to fit models, preventing high-concentration data from dominating the sum-of-squares error. A nonlinear regression method that accounts for measurement error in *C*_w_ has been developed.^72^ Solute depletion limits need to be taken seriously owing to measurement errors in *C*_w_, the correlation between errors in *C*_w_ and the calculated *q*, and the limits of detection. The “20%–80%” rule-of-thumb is recommended. The solid-to-liquid ratio should be adjusted to achieve at least 20%, and preferably 50% sorption to minimize errors in delta *C*_w_ between sample and control, which is used to calculate *q*.^73^ Likewise, solute depletion should be kept < ∼ 80% to elude detection limits, especially toward the low end of the isotherm, where *C*_w_ is smaller and the error in *C*_w_ is larger. Solids concentration *per se* has no effect on the isotherm, but solids at varying concentrations may differentially remove background solutes (e.g., DOM, other TrOCs) present in waters of interest, which could influence TrOC sorption. In a fresh column, both DOM and TrOC concentrations rise as breakthrough is approached. In most cases, DOM reaches breakthrough sooner than TrOCs. Consequently, dissolved TrOCs will experience a wide range of DOM concentrations until TrOC breakthrough. Therefore, it is beneficial to compare sorption on sorbents fully pre-equilibrated at the two extremes, pure water and background solution, the latter using a multistep fill-centrifuge-decant-refill process. The background solution composition should represent the treatment scenario. Sorption of ionic or ionizable TrOCs will be especially sensitive to pH, ionic strength, and the identities of background cations and anions. For this reason, avoid buffers unless sorption is shown to be independent of buffer concentration. Instead, adjust pH with NaOH or HCl. In simple systems with only a single solute, equilibrium isotherms are known to be consistent over broad ranges of sorbent-to-solute ratios. In these cases, varying solute concentration with a constant sorbent dose or varying sorbent dose with a constant solute concentration should yield similar results. While it is possible to extract isotherm parameters from miscible displacement (column elution) experiments, the drawbacks of that approach are serious: the need to use sufficiently low flow rates to permit the assumption of sorptive equilibrium at the particle level, and the need to carry out injections at multiple concentrations if the isotherm is anticipated to be nonlinear.

However, such simple systems typically do not represent environmental conditions with complex water chemistry and competition from other solutes, including DOM. For example, isotherms conducted with constant sorbent dose and varying solute concentration in the presence of DOM can appear linear, while experiments conducted under the same conditions but with constant solute concentration and varying sorbent dose have been observed to produce nonlinear isotherms.^52,74^ Confounding this observation is the apparent independence of the normalized solute breakthrough curve (C_out_/C_in_ plotted as a function of bed volumes of water treated) on inlet concentration in flow-through systems. While this independence is consistent with linear isotherms under ideal single-solute conditions, the displacement of weakly adsorbing solutes by more strongly adsorbing solutes is not captured by the linear isotherm model. The apparent linearity and concentration-independent breakthrough were due to competing substances in the water, mainly DOM, which could be modeled with IAST.^52,75^

In batch experiments, mix as gently as possible to minimize particle breakup; end-over-end tumbling at 1 cycle per minute is sufficient for overnight or longer equilibration. Magnetic stirring is a notorious cause of particle grinding. This raises the question of the equilibration period. It is essential to capture the equilibrium or static condition as closely as possible. This may require much longer contact times than those experienced in the field with the TrOC-sorbent. Carbonaceous sorbents are renowned for displaying multiphasic kinetics, with fast, slow, and exceedingly slow phases. This is true even of colloidal-size AC particles.^76^ In general, for carbonaceous sorbents and soils, diffusion is slower in smaller, higher-affinity pores and faster in larger, lower-affinity pores. Thus, sorption distribution plots will shift upward with equilibration time inversely with concentration, displaying increasingly greater nonlinearity as equilibrium is approached.^77,78^ In preliminary experiments to determine the appropriate equilibration time, it is advisible to follow the uptake rate at the lowest practicable concentration position on the isotherm. To determine the adsorption capacity of GAC, the material is typically first ground using a mortar and pestle to obtain the 100 × 200 US mesh fraction (∼105 μm geometric mean diameter) or smaller. At this particle size, adsorption equilibrium is typically achieved within about 3 weeks.^79,80^ At long equilibration times, it may be necessary to prevent TrOCs biodegradation. No sterilant or inhibitor is ideal. The best of numerous methods tested on soil were twice- or three-times autoclaving, propylene oxide, γ-irradiation, and mercuric chloride,^78^ but all may alter sorbent properties. Azide is a poor inhibitor^78^ and, in studies of ionic/ionizable contaminants, should be avoided.

## Experimental Design, Interpretation, and Modeling of Sorption Kinetics

Sorption kinetics are an important component of assessing the utility of a sorbent. Mass-transfer processes relevant to sorbate uptake include film diffusion, aqueous pore diffusion, and surface diffusion, as shown in Figure 1. While solute adsorption to the solid phase from the aqueous phase is typically assumed to occur nearly instantaneously, the rate of solute mass transfer through the liquid film or sorbent pores to a sorption site is often limiting relative to the adsorption rate, particularly for porous sorbents.^74,81–83^ Film diffusion refers to the transport of the solute from the bulk solution across a thin, stagnant boundary layer (film) to the external surface of the sorbent. Aqueous pore diffusion refers to the transport of a solute through internal solution-filled pores of a sorbent. Surface diffusion refers to the transport of sorbed solutes along the external and internal surfaces of a sorbent. The properties of the TrOC and sorbent greatly impact mass-transfer rates and, consequently, the overall removal rate.^84^ For TrOCs in porous sorbents, mass-transfer limitations often govern breakthrough behavior and treatment performance, particularly under flow-through conditions. Understanding the kinetics of solute removal is essential for the design and deployment of sorbents in natural environments and engineered treatment systems.

**Figure 1:**
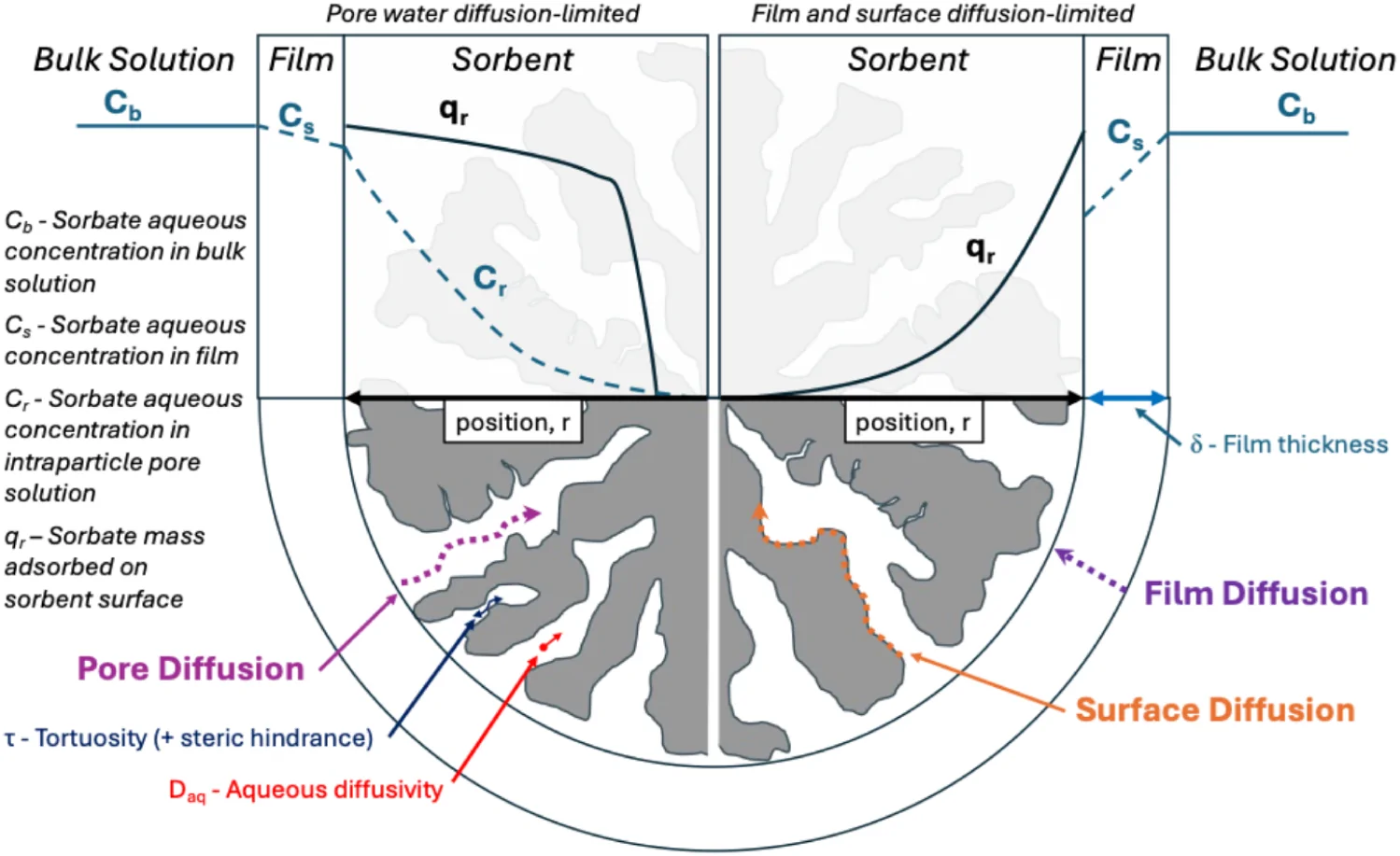
This diagram shows a schematic representation of concentration profiles representative of a pore water diffusion limited system (top left) and a film and surface diffusion limited system (top right), and of various mass-transfer processes of sorbate uptake relevant parameters (bottom). This diagram is adapted from the concentration profiles from Sontheimer et al.^137^

Various sorption kinetic models, ranging in complexity from pseudo-first-order and second-order equations to mechanistic mass-transfer models, that are commonly used are summarized in Table 2. Pseudo-first-order and second-order models have been used to describe the apparent mass-transfer kinetics.^85^ While pseudo-first-order kinetics can have some mechanistic basis, particularly under conditions where external film diffusion is rate-limiting, pseudo-second-order kinetics are entirely empirical.^86^ These models generally are not transferable across systems or operating conditions and are dependent on the exact experimental conditions used.^87^ While convenient for extrapolating sorption curves for batch experiments, they are not useful for predicting TrOC breakthrough curves in flow-through systems or for comparing kinetic parameters across different experimental conditions. Linear driving force and two-site/dual-domain models represent empirical, semimechanistic approaches that better capture the time-dependent approach to equilibrium by incorporating simplified representations of mass-transfer limitations, while still maintaining a tractable mathematical form. However, these models remain lumped approximations that do not explicitly resolve individual mass-transfer pathways (e.g., film, pore, or surface diffusion), and therefore their parameters are only partially transferable across systems with differing hydrodynamic or physicochemical conditions. Mechanistic models can include film, surface, and aqueous pore diffusion mass-transfer processes. These models are based on first-principles of mass transfer and explicitly represent where transport occurs, in which phase it occurs, and the driving force.^88^ Dimensionless numbers, such as those summarized in Table 3, are useful for identifying mass-transfer process efficiency. The Peclet number characterizes external transport within the porous media bed (i.e., advective flow compared to diffusive flow). The Sherwood number assesses the efficiency of external mass transfer (i.e., fluid mechanics near the sorbent particle). The Biot Number, representing the ratio of external film mass transfer to internal pore diffusion, is particularly useful for assessing whether internal or external mass transfer is limiting.^89^ Depending on the rate-limiting step, a film diffusion model or an intraparticle diffusion model (pore or surface diffusion model) can be selected to reduce model complexity.

**Table 2:** Common Mass-Transfer Modeling Approaches Used to Describe Solute Sorption in Porous Media Systems

Modeling Approach	Governing Concept	Mass-Transfer Parameters	Physical Meaning of Parameter(s)	Dominant Process Represented
Pseudo-first Order (PFO)	Removal rate proportional to deviation from equilibrium	*k*_1_	Empirical lumped parameter	Lumped (film, pore, surface)
Pseudo-second Order (PSO)	Removal rate is proportional to square of available capacity	*k*_2_	Empirical lumped parameter	Lumped (film, pore, surface)
Linear Driving Force (LDF)	Lumped approximation of diffusion-controlled uptake	k_LDF_	Roughly approximates diffusion	Primarily intraparticle diffusion
Two-site/dual-domain model	Instantaneous + rate-limited sorption domains	*k*_mt,1_, *k*_mt,2_, site distribution	Lumped rate constant and site distribution	Diffusion into more and less accessible domains
Film diffusion model	External boundary layer controls transfer	*k*_f_	Mechanistic (hydrodynamics-dependent)	Film diffusion
Intraparticle pore diffusion model	Diffusion within particle pores controls uptake	D_p_	Mechanistic (effective diffusivity)	Pore diffusion
Intraparticle surface diffusion model	Diffusion along sorbed phase on surfaces	D_s_	Mechanistic (surface mobility)	Surface diffusion
Dual-resistance (film + pore diffusion)	Combined external and internal resistances	*k*_f_, D_p_, D_s_	Fully mechanistic	Film diffusion, pore diffusion, and surface diffusion

**Table 3:** Key Dimensionless Numbers for Diagnosing Mass-Transfer Limitations^a^ v

Dimensionless Number	Definition	Physical Meaning	Use	Interpretation
Biot Number	$\mathrm{Bi}=\frac{k_{f}L}{D_{p}}$	Ratio of external (film) to internal (pore) mass transfer rates	Relative importance of film or intraparticle diffusion	Bi ≪ 1: film diffusion limiting
				Bi ≈ 1: mixed
				Bi ≫ 1: pore diffusion limiting
Pecelet Number	$\mathrm{Pe}=\frac{vL}{D}$	Ratio of advective to diffusive transport	Whether flow is dominated by flow or diffusion	Pe ≪ 1: diffusion dominated
				Pe ≈ 1 – 10: mixed
				Pe ≫ 100: advection dominated
Damköhler Number	$\mathrm{Da}=\frac{\mathrm{kL}}{v}$	Ratio of reaction/sorption rate to transport rate	Whether system is kinetically limited or equilibrium controlled	Da ≪ 1: kinetically limited
				Da ≈ 1: mixed
				Da ≫ 1: near equilibrium
Sherwood Number	$\mathrm{Sh}=\frac{k_{f}L}{D}$	Dimensionless mass transfer coefficient	Importance of external mass transfer (film resistance)	Low Sh: strong film resistance
				High Sh: weak film resistance

a *k*_f_ is the external film mass-transfer coefficient; L is the characteristic length scale; D_p_ is the intraparticle (pore) diffusion coefficient; *v* is the characteristic fluid velocity; D is the molecular diffusion coefficient in the fluid phase; and k is the reaction or sorption rate constant.

For TrOCs in porous carbonaceous sorbents such as GAC and biochar, intraparticle diffusion is typically rate-limiting relative to film diffusion.^90,91^ Within intraparticle mass-transfer processes, surface diffusion is often rate-limiting in single-solute systems involving GAC, while pore diffusion tends to be rate-limiting in GAC columns in which TrOC sorption occurs in the presence of DOM.^84^ For ion exchange resins, kinetics are typically governed by intraparticle macropore or gel diffusion within the polymer matrix.^92,93^ However, film mass transfer can become important for ionic TrOCs, such as PFAS.^94^ Little information on the rate-limiting step is available for novel sorbents, such as MOFs and COFs, partly because appropriate experimental data to discern mass-transfer limitations are lacking and partly because mechanistic models are not routinely applied. When the dominant transport mechanism is correctly identified, and the model is appropriately parametrized, mechanistic models offer the greatest predictive value across scales and treatment configurations.

Sorbents often exhibit substantial heterogeneity in particle size, pore structure, and surface accessibility. Such sorbent properties impact sorbate mass-transfer rates and equilibrium time scales.^84^ For instance, particles differing in radius by 10^2^ can differ in the time it takes to reach 95% of equilibrium by a factor of 10^4^ because diffusion rates are inversely proportional to the square of the sorbent particle radius.^83^ Multiple-particle class models better represent long-term sorption rates than single-particle class models.^95^ The solution to diffusion models typically requires numerical, e.g., finite-difference or finite-element, algorithms, which are available in open-source models.^96^ For polymers and possibly MOFs, the diffusion coefficient may be intrinsically concentration-dependent owing to effects of the sorbate on sorbent properties (e.g., plasticization). In that case, the function relating diffusivity to concentration must be known, but no suitable theory has been established for this relationship.^97,98^ Competing sorbates may enhance the diffusivity of the contaminant of interest by preferentially occupying relatively “tight” sites, leaving the contaminant to diffuse through “looser” sorption domains. Carbonaceous and ion exchange sorbents are usually assumed to be isotropic (uniform network of pores of uniform size) for modeling purposes. Nonlinear monoporous diffusion models, such as the Homogeneous Surface Diffusion Model (HSDM), Pore Diffusion Model (PDM), and Pore Surface Diffusion Model (PSDM), are commonly used.^96^ In these models, diffusion occurs in the porewater of isotropic particles or particle aggregates, and sorption at the local level is nonlinear and assumed to be at equilibrium. But carbonaceous sorbents are inherently anisotropic and exhibit multiple kinetic phases. Biporous diffusion models, such as the Microspheres-in-Macrospheres model,^99^ the Macrosphere-with-In-Series-Cylindrical-Macropore-and-Micropore Networks model,^99^ or the Branched Pore Kinetic (BPK) model,^100,101^ have been considered, but require knowledge (or estimation) of the physical properties and equilibrium sorption parameters of each pore domain and a definition of the boundary condition between them.

### Difficulties in Using Batch Experiments to Estimate Mass-Transfer Coefficients

Determining the kinetic parameters necessary for predictive modeling of solute removal in a flow-through system is challenging. Kinetic data are often obtained from bench-scale batch experiments in continuously mixed vessels containing a known concentration of solute, mass of sorbent, and background aqueous matrix. Vessels may be subsampled or sacrificially sampled over hours or days. While batch experiments are often simpler to design and execute, they often suffer from fundamental limitations arising from inherent differences between batch and column systems. These differences can lead to obtaining kinetic parameters that either over- or underpredict TrOC removal in column systems.^102^ Beyond the considerations discussed above for equilibrium isotherms, the following outlines additional challenges relevant to assessing sorption kinetics.

#### Constant Mixing

Similar to batch equilibrium experiments, batch kinetic experiments are typically conducted under constant mixing, which helps ensure that solute concentrations are uniformly distributed in batch reactors and improves reproducibility. However, mixing reduces the external boundary layer thickness, inflating the film mass-transfer coefficient and suppressing film mass-transfer limitations.^103^ Thus, the system is biased toward intraparticle diffusion mass-transfer limitation. While intraparticle diffusion is typically the rate-limiting mass-transfer process for carbonaceous sorbents, this is not universally true for all sorbents. For example, ion exchange processes can be limited by film diffusion.^94^ Moreover, overly vigorous mixing can produce finer-grain material from granular sorbents, greatly accelerating TrOC removal rates and skewing the resulting kinetic parameters. Such artifacts can be avoided by using differential column batch reactors (DBCRs) to determine intraparticle diffusion coefficients for granular sorbents.^104^

#### Sorbent Dose

In kinetic batch experiments, sorbent doses should be selected to preserve sufficient aqueous-phase TrOC concentrations throughout uptake so that concentration-time profiles can resolve the governing mass-transfer processes. Excessive sorbent doses can cause rapid solute depletion, compressing uptake curves and obscuring distinctions between film diffusion, intraparticle diffusion, or other rate-limiting processes. The sorbent dose should be selected such that the equilibrium sorbate concentration is at least an order of magnitude above detection limits and ideally 40–60% of the initial concentration to enable accurate mass balance determinations. Additionally, the initial sorbate concentration should be as representative of field conditions as possible while allowing adequate detection and quantification of aqueous equilibrium concentrations. Strategies for maintaining adequate sorbate concentrations include adjusting the solid-to-liquid ratio. Ensuring adequate analytical quantification of sorbate concentrations is essential for determining scalable kinetic parameters, particularly when empirically fit to batch uptake curves.

#### Competing Solute Concentrations over Time

Sorbents in batch tests are exposed to a finite mass of competing solutes (such as DOM), and their aqueous concentrations can decrease over time. In a column experiment, however, sorbents are exposed to a continuous influx of competing solutes. Competing solutes can displace the target solutes, particularly in cases when the target solute adsorbs more weakly than the competing solutes.^105^ Continued inflow of DOM in flow-through systems can lead to sorbent fouling, such as by pore-blockage or preloading of sorption sites ahead of the TrOC mass-transfer zone, which is substantially greater than what would be observed in batch tests.^84^

### Limitations in Applicability of Kinetic Parameters Obtained from Batch Experiments

When applied to batch kinetic data, fully mechanistic models permit the independent determination of kinetic parameters (e.g., film mass-transfer coefficients, effective pore and/or surface diffusivities). However, the resulting parameters do not fully capture dynamic processes that occur in column reactors over long periods of time, such as sorbent fouling by DOM and/or inorganic precipitates, as well as the beneficial or detrimental effects of biofilm growth, which is often inhibited in batch tests to control for TrOC biodegradation and ensure sorption as the only removal mechanism. Batch kinetic tests are often too short to establish biological growth at a level observed in pilot- or full-scale columns. Batch kinetic tests can therefore overestimate sorption rates of recalcitrant TrOCs by preventing fouling from biofilm growth, but may underestimate overall removal of biodegradable TrOCs when biofilms contribute to TrOC degradation in pilot- or full-scale column systems.^94^ Additional parameters are often needed to obtain fitted, system-specific parameter values that describe, for example, temporal changes in equilibrium parameters as well as film mass-transfer and intraparticle diffusion coefficients.^106–108^ Therefore, while these mechanistic models are, in practice, somewhat empirical, they are the best approaches available for describing sorption kinetics and are far superior to pseudo first- or second-order models. As such, kinetic parameters fit from batch experiments in which sorption is isolated as the sole removal mechanism may not be as useful as kinetic parameters that may combine other processes such as media fouling. Ideal sorption kinetic parameters obtained for a single solute in deionized (DI) water are not useful for predicting removal of a solute in a complex matrix with competing solutes, including DOM.

### Relevance of Batch Experiments

Despite the challenges mentioned above, batch kinetic experiments can provide useful data in certain instances. Specifically, batch kinetic experiments are relevant for powdered sorbents (such as PAC – 90% passing 45 μm mesh) that move with the water and equilibrate with the effluent concentration of a process. Batch kinetic experiments are a reasonable starting point for establishing how a sorbent will perform and gaining insight into mass-transfer limitations. In such cases, it is recommended to perform equilibrium experiments first, followed by batch kinetic experiments. When comparing sorbents, kinetic experiments should be performed with particles of the same or very similar size, especially when intraparticle diffusion is the rate-limiting mass-transfer step. To derive kinetic parameters for granular adsorbents that are relevant for predicting sorbent performance in full-scale columns, experiments must be intentionally designed to identify limiting mass-transfer processes and determine relevant mass-transfer parameters. Experimental approaches that have been used most successfully include short-bed adsorber (SBA) tests and rapid small-scale column tests (RSSCTs).^109,110^

### Suggested Column Experimental Approaches for Determining Scalable Mass-Transfer Parameters

Column experiments are often needed to assess sorption kinetics of granular sorbents that equilibrate with the column influent concentration in matrices with more complex water chemistry. They are particularly advantageous with granular sorbents because fines generated in batch reactors can influence kinetic parameters. Column test approaches that minimize the time needed to determine mass-transfer parameters include RSSCTs and SBA tests. In each of these strategies, the underlying objective is to generate a breakthrough curve for estimating mass-transfer parameters using a mechanistic model, such as the pore-surface diffusion model. The resulting parameters can then be used to predict sorbent performance under other conditions, including different column configurations, influent TrOC concentrations, and scales.

#### Rapid Small-Scale Column Tests

An RSSCT is a scaled-down column test that uses smaller particle sizes and bed dimensions to accelerate sorption processes.^111^ RSSCTs are designed by applying the principle of similitude to the dimensionless form of the pore-surface diffusion model. The contact time and flow velocities are scaled to preserve diffusion time scales and hydrodynamic conditions, ensuring they represent full-scale systems. RSSCTs can treat tens of thousands of bed volumes in days to weeks while minimizing the influent water volume required and the effluent generated.^94^ The two main RSSCT design approaches assume that intraparticle diffusivity (1) does not vary with sorbent particle size (constant diffusivity RSSCT or CD-RSSCT) and (2) varies linearly with sorbent particle size (proportional diffusivity RSSCT or PD-RSSCT).^111,112^ In the RSSCT approach, it is assumed that sorption isotherms and mass-transfer mechanisms are consistent across particle sizes, and that the contact time (often expressed as empty-bed contact time) is properly scaled. Limitations of RSSCTs include that RSSCTs may not capture fouling or long-term aging,^113^ and their application to novel sorbents can present additional challenges, as discussed in the following section. As such, it is recommended to conduct pilot studies at scale to confirm RSSCT findings.

#### Short-Bed Absorber Tests

An SBA test is a column test that employs a short, packed bed that does not fully contain the TrOC mass-transfer zone at typical hydraulic loading rates. As a result, partial TrOC breakthrough occurs immediately after starting the experiment (e.g., C_out_/C_in_ ∼ 0.3–0.6).^114,115^ The immediate level of breakthrough is directly linked to the film mass-transfer coefficient, while later data are sensitive to changes in parameters describing intraparticle diffusion.^81,114–116^ SBA test data are useful for parametrizing dual-resistance mass-transfer models, in which both film diffusion and intraparticle diffusion resistance are quantified.^52^ This approach is considered the most rigorous for determining film-diffusion and intraparticle-diffusion parameters.^109^ Additionally, various conditions can be easily probed, such as changes in particle size or hydraulic loading rate. However, SBA tests also suffer from limitations of not capturing sorbent fouling by background water matrix constituents such as DOM, which will occur in full-scale systems operating for long periods of time.

#### Alternative Approaches

While RSSCTs and SBA tests are most efficient for establishing reproducible mass-transfer parameters in short time frames, other experimental approaches may be needed depending on the application. For example, challenging water matrices and high fouling potential may require longer-term experiments, whereas variable flow regimes, intermittent flows, or unsaturated conditions may require a full-scale bed design to capture hydrodynamic complexities.^23,117^ Alternative strategies for inducing faster breakthrough under full-scale conditions include reducing sorbent content in columns.^118^ In such cases, using 1–3% w/w sorbent content when mixed with inert material, such as sand, allows for a faster breakthrough curve that can be assessed over weeks or months.^119,120^ Using a full-scale system is more time and resource-intensive but does not require scale-up. Additionally, gravity-driven configurations allow for monitoring hydraulic conductivity.^22^ Sample ports at different bed depths can also be used to assess breakthrough curves along the column, which can be useful for studying solutes with varying removal performance, for evaluating Empty Bed Contact Time (EBCT) effects, and for generating multiple breakthrough curves that can be used to parametrize models.^22,121^ Finally, the plethora of column data available in the peer-reviewed literature lends itself to the development of machine learning models that can identify key factors affecting TrOC removal from complex water matrices in bench-, pilot-, and full-scale sorption processes.^122^

## Evaluation and Transition of Novel Sorbents to Scaled Sorption Processes

Recent advances in materials science have enabled the rapid development of novel sorbents for selectively removing TrOCs from water. These materials leverage distinct design strategies, including tunable surface chemistry, engineered pore structures, and molecular recognition mechanisms, to enhance affinity and selectivity at the molecular level. Selectivity, as we use the term, does not distinguish among or reflect the contributions of individual sorption mechanisms; it is an empirical measure of competitive performance in complex matrices. For example, cyclodextrin-based polymers use host-guest complex formation to preferentially capture hydrophobic organic contaminants,^16^ while molecularly imprinted polymers (MIPs) introduce template-defined binding sites that recognize target molecules.^123,124^ MOFs,^125^ MOF-derived porous carbon,^63^ and ionic liquid-functionalized MOFs^126^ utilize high surface areas and well-defined microporosity to maximize sorption capacity, with the addition of tailored surface functionalities for specific sorbate-sorbent interactions. In parallel, carbonaceous sorbents, including modified biochars and ACs, have been extensively explored as alternatives to their unmodified counterparts, with functionalization strategies introduced to enhance sorption by enabling interactions beyond the predominantly hydrophobicity-driven behavior of conventional carbon materials.^18^ By incorporating heteroatom- or metal-based functional groups and developing hierarchical pore structures, these materials introduce coupled mechanisms, such as π-π interactions, electrostatic attraction, hydrogen bonding, and surface complexation, enhancing selectivity for targeted TrOCs.^15,17,127^

Novel materials span a wide range of technological maturity, from early stage, proof-of-concept systems, such as MOFs and engineered polymers, to more mature platforms, such as modified carbon-based sorbents. As a result, the dominant challenges also evolve: early stage materials require deriving sorption parameters with physically meaningful information to inform mechanistic understanding, performance prediction, and technical scale-up, whereas more mature sorbents are increasingly constrained by consistency, quality control, and integration into scalable production and treatment systems. However, current evaluation practices remain largely misaligned with these needs. Laboratory studies frequently report promising removal performance under controlled but oversimplified conditions that do not adequately capture the thermodynamic and kinetic constraints encountered in practical treatment systems. While these studies provide useful insight into intrinsic sorbate-sorbent interactions, the resulting performance metrics often depend strongly on experimental design and therefore lack a consistent basis for comparison across studies or extrapolation to realistic treatment scenarios.

Addressing this gap requires experimental approaches that prioritize generating mechanistically meaningful and transferable data. Sorption experiments should be designed under conditions that enable results to be interpreted, compared across materials, and linked to expected treatment performance rather than to a specific experimental setup. This includes accounting for equilibrium, competitive sorption behavior, mass-transfer limitations under relevant hydrodynamic conditions, realistic water matrices, environmentally relevant concentrations, and solid-to-liquid ratio, as well as reporting parameters with clear physical meaning that support predictive modeling and scale-up. Such data sets are also essential for leveraging emerging computational and data-driven approaches, including machine learning, to identify structure-performance relationships and guide the rational design of next-generation sorbents. In the following sections, we highlight key considerations in novel sorbent evaluation, spanning early stage screening under environmentally relevant conditions, translation of powdered materials into scalable process configurations, validation of particle-size and mass-transfer scaling relationships, and ultimately the generation of standardized data sets to support data-driven sorbent design.

### Designing Batch Experiments for Early-Stage Sorbent Evaluation

Many studies report novel sorbents that exhibit remarkable performance in removing one or more TrOCs from water under idealized experimental conditions.^16,128^ These results are not always transferable to engineered treatment systems. Some considerations are particularly important when designing experiments for early stage evaluations of a novel sorbent to generate data that are both mechanistically meaningful and transferable.

A key consideration is the selectivity of target TrOCs relative to co-contaminants and background water matrix constituents. In ion exchange systems, selectivity is routinely quantified using selectivity coefficients that describe competitive partitioning between ions. In contrast, sorption studies for TrOCs rarely explicitly quantify selectivity, and removal is often reported as percent removal or sorption capacity for a single contaminant, making it difficult to determine whether the observed removal reflects intrinsic affinity or simply the absence of relevant competitors. While sorption processes are governed by multiple interacting mechanisms that complicate the definition of a universal selectivity coefficient, there remains a clear need for quantitative metrics that capture competitive sorption behavior and enable comparison across materials under representative conditions. In the absence of standardized selectivity metrics, experiments should, at a minimum, be conducted in representative water matrices, including groundwater, surface water, drinking water, or wastewater, or in synthetic representations of one or more of these water types, evaluated over a range of representative pH levels and temperatures. Some novel sorbents can exhibit high selectivity for target TrOCs in the presence of some types of matrix constituents. For example, cyclodextrin-based polymers that rely on host–guest complex formation between target TrOCs and the interior cavity of the cyclodextrin are selective for TrOCs of a certain molecular volume over DOM and other larger organic molecules.^129^ Surface-modified carbon materials, particularly metal-modified biochars, have demonstrated enhanced affinity toward anionic PFAS through electrostatic interactions between positively charged surface sites and PFAS head groups, with performance maintained in field-collected stormwater characterized by high turbidity and elevated organic carbon.^15^ Evaluating sorption in representative matrices, therefore, provides a more reliable basis for assessing whether laboratory-scale observations are transferable to treatment systems.

A second consideration is the selection of sorbent dose and contaminant concentration. Early stage screening of novel sorbents emphasizes identifying intrinsic affinity and practical feasibility under representative conditions before more detailed isotherm and mass-transfer characterization is warranted. In particular, most early stage evaluations rely on batch experiments in which the sorbent, typically a fine powder, is introduced into a reactor containing the target TrOC(s) in a given water matrix. Removal efficiency is strongly dependent on both sorbent dose and TrOC concentration, and high sorbent loading or relatively low TrOC concentrations can lead to apparently complete removal even when sorption affinity is modest. Consequently, commonly reported metrics such as percent removal are difficult to interpret and cannot be directly compared across studies. In addition, percent removal is typically derived under equilibrium conditions, which implicitly assumes that all contaminants have reached equilibrium with sorbents despite potentially large differences in mass transfer, further complicating interpretation and comparison. It is also essential to demonstrate performance at the lowest feasible sorbent doses under environmentally relevant concentrations. This serves two purposes. First, it provides insight into practical feasibility, as novel sorbents must deliver improved performance relative to conventional materials to justify potential increases in material cost and life-cycle management requirements. Second, it enables quantification of intrinsic sorption properties; when removal is complete due to excessive sorbent loading, key parameters such as *K*_d_ cannot be determined. Although sorption isotherms are often constructed to characterize sorption behavior, parameters such as Langmuir maximum capacity (*Q*_L_) are typically derived from data collected at elevated concentrations and extrapolated beyond environmentally relevant regimes. For TrOCs, greater attention should be given to the low-concentration region of the isotherm, where contaminants are typically present at low levels in the presence of excess background constituents. Practical feasibility should be evaluated by balancing performance with life-cycle costs, not material costs alone. Regeneration or reactivation is attractive because it enables sorbent reuse and reduces replacement and disposal requirements, although a detailed evaluation of regeneration strategies is beyond the scope of this review and is discussed elsewhere.^130^ The economic feasibility of novel sorbents may evolve rapidly as production scales, supply chains mature, and life-cycle management strategies advance. As a practical starting point for early stage screening, sorbent doses on the order of ∼ 1–10 mg L^–1^ in relatively clean water matrices and up to ∼ 10–100 mg L^–1^ in more complex matrices provide a useful basis for comparison with conventional sorbents but may need to be adjusted depending on sorbent affinity to ensure that the equilibrium sorbate concentration remains at least an order of magnitude above detection limits. Under these conditions, sorption behavior is more appropriately described using affinity-based parameters such as *K*_d_, evaluated in the linear region of the isotherm or within environmentally relevant concentration ranges, whereas full high-concentration isotherms may be more informative for mechanistic interpretation and transport model development.^52^

A third consideration is benchmarking performance against state-of-the-art sorbents used in current practice. Demonstrating removal of a target TrOC alone is insufficient to establish the value of a novel sorbent; performance should be evaluated relative to existing sorption technologies under comparable conditions to determine whether the new material offers a meaningful advancement in sorption affinity, selectivity, or operational robustness relative to conventional sorbents used in practice. Benchmarking experiments should therefore include conventional sorbents tested in parallel under identical conditions, particularly materials widely used in practice, such as AC. These comparisons should be conducted under the same environmentally relevant conditions and at comparable sorbent doses and TrOC concentrations (chemical activity) to ensure meaningful interpretation. Differences in particle size and experimental configuration should also be considered, as these factors influence mass transfer and apparent sorption performance; where necessary, materials should be evaluated in similar physical forms (e.g., powdered or granular), and representative particle size metrics should be reported (e.g., *d*_10_, *d*_50_, and *d*_90_). When possible, grind and sieve larger particles to match the particle size distribution of finer adsorbents. In addition, rigorous physicochemical characterization of sorbents is essential for meaningful comparisons across materials. Even when experiments use the same sorbent dose, differences in properties such as surface area, pore size distribution, and surface chemistry can strongly influence sorption behavior and complicate interpretation. For example, biochars can exhibit sorption hysteresis and pore accessibility limitations that lead to discrepancies in surface area measurements. Thus, multiple lines of evidence and rigorous data quality control are needed to ensure reliable interpretation and comparison across materials. Including conventional sorbents in these experiments not only strengthens claims about the performance of novel materials but also generates comparative data sets that improve broader understanding of sorption behavior across different sorbent classes.

### Designing Experiments to Predict Performance of Novel Sorbents in Scaled Treatment Processes

The next stage of evaluation for promising novel sorbents is process-based experiments that evaluate performance under specific operational conditions. These evaluations, like those used for conventional sorbents, include batch treatability studies, RSSCTs, and pilot-scale column tests. However, novel sorbents often present distinct challenges related to particle morphology, mass-transfer behavior, materials processing, and scale-dependent changes in sorption performance.

Most novel sorbents are synthesized as powders with particle sizes in the range of one to tens of microns with varying degrees of porosity. Particle size can strongly influence mass-transfer behavior and apparent sorption performance. This makes batch experiments relatively easy, but well-performing novel sorbents intended for scalable use in flow-through porous media will require design modifications to produce particles of the desired size and morphology. Strategies for preparing granular materials include grafting onto an inert substrate,^131^ postsynthetic granulation,^132^ or various types of suspension polymerization to directly produce spherical beads of a desirable size. Any process selected to generate larger sorbent particles is expected to change the physical properties of the sorbent in different ways. In other words, the way that the performance of the sorbent scales with particle size is a function of both the sorption mechanisms and the accessibility of the sorption sites in the scaled particle.^9^ For example, grafted materials generally have lower sorption site densities on the scaled sorbent particle due to the presence of the inert substrate, whereas particles synthesized by means of suspension polymerization may maintain accessibility to sorption sites in ways that are more analogous to the powdered sorbent. Notably, benchmarking is challenging because most conventional sorbents are acquired as larger particles and pulverized to generate powders, whereas novel sorbents are most often synthesized as powdered materials that need to be modified to generate larger particles for scaled applications. As a result, sorbent performance will likely scale differently across particle sizes and, in practice, any sorbent that outperforms another in powdered form may show the opposite trend as larger particles. For example, powdered AC performs significantly better as an adsorbent with respect to sorbate uptake relative to the same mass of GAC at non-equilibrium conditions.^52,100,133^ The same might not be true for some novel sorbents whose performance might be worse than powdered AC at small particle sizes, but much better than GACn at larger particle sizes.

Sorbent performance as a function of particle size is an important confounding issue that limits the rapid transition of novel sorbents into scaled processes. Although the preceding sections discussed how to determine equilibrium and mass-transfer parameters, another challenge for novel sorbents is determining whether these parameters remain transferable across particle sizes. Ideally, experiments would be performed at varying particle sizes to establish scaling relationships among these parameters to enable the development of theory on the scaled performance of novel sorbents. Although approaches such as RSSCTs have been rigorously established for conventional sorbents,^94,134^ they are often extended to novel sorbents without fully validating whether the underlying mechanistic and scaling assumptions remain applicable. Careful experimentation to validate parameter transferability across particle sizes, flow conditions, and sorbent forms will be essential for advancing RSSCTs and other approaches for novel sorbents. Without fully validated mechanistic models to support predictions of scalable performance for novel sorbents, pilot-scale data across multiple operating conditions may be needed to validate scaling assumptions. Because pilot-scale studies require significant cost and effort, they are considered late-stage performance evaluations to support implementing novel sorbents in engineered processes. At this stage, approaches such as RSSCTs should ideally be used to predict pilot-scale TrOC breakthrough, with agreement between RSSCT and pilot-scale breakthrough behavior providing an important validation of the underlying scaling assumptions. Further, novel sorbents must be evaluated not only in their as-synthesized condition and morphology, but also under aged or field-relevant conditions to assess long-term performance, structural stability, and resistance to fouling or performance loss.

While the preceding section focuses on packed-bed systems relevant to drinking water and wastewater treatment, it is also important to recognize that some novel sorbents, such as modified carbons and carbon-based composites, can be deployed as amendments within heterogeneous media such as aquifers or sediment beds.^21^ In these systems, the sorbent is distributed within a porous matrix rather than used as a uniform packed bed and is subjected to variable flow conditions and complex water chemistry. The performance of conventional sorbents, such as biochar and AC, in these amendment-based systems has been described using transport models that couple advection, dispersion, and intraparticle diffusion-limited sorption.^22,23,44^ Similar modeling frameworks can be extended to evaluate novel sorbents under these conditions. However, it is important to recognize that the parameters derived from such models require careful interpretation. For example, the apparent intraparticle tortuosity term is a composite parameter that reflects not only pore-scale diffusion but also other unresolved transport limitations and therefore may not be directly transferable across materials or system configurations. In addition, water chemistry effects, including dissolved organic matter and ionic strength, are often implicitly lumped into fitted parameters, limiting the model’s predictive capability under changing conditions. Decoupling these effects and improving the mechanistic basis of model parameters remain important areas for future work.

Another challenge for novel sorbents in these systems is the consistency of material properties and the translation of modifications into scalable production. Material property inconsistency has been a key barrier to the broader adoption of biochar, as variability in feedstock and thermochemical conversion conditions can lead to substantial differences in pore structure, surface chemistry, and sorption performance. Similar challenges are expected for other novel sorbents, particularly those involving functionalization or composite synthesis, where small changes in preparation conditions may significantly alter performance. As a result, ensuring modified materials can be produced at scale with consistent properties while maintaining performance remains critical for deployment. Established practices for conventional sorbents such as AC, including ASTM-based standards for material specification and testing, have played an important role in enabling consistent production and widespread adoption. Developing analogous frameworks for novel sorbents that account for their greater structural and compositional variability will be essential to facilitate reproducibility and integration into real-world applications.

### Material Design and Discovery Enabled by Data and Artificial Intelligence

Machine learning and data-driven approaches to sorbent design and discovery have gained increasing attention, driven in part by the growing body of sorption data available for TrOC-sorbent systems.^135,136^ However, the heterogeneity of these data sets, arising from differences in experimental design, water matrices, contaminant concentrations, and reporting metrics, limits their comparability and reduces their value for guiding material design. This heterogeneity reflects not only inconsistencies in experimental protocols but also the widespread use of simplified conditions that do not capture the coupled effects of water chemistry, competing solutes, and transport limitations that govern performance in practice. The experimental practices advocated throughout this review, including environmentally relevant conditions, benchmarking against conventional materials, and extraction of mechanistically grounded parameters, can help generate more consistent and interpretable data sets. When paired with descriptors of contaminant and sorbent properties, such data sets provide the foundation for identifying structure-performance relationships and for training data-driven models capable of guiding rational sorbent design and screening large libraries of candidate sorbent structures. Advancing data-enabled sorbent design will therefore require community-level standards for data reporting and parameter extraction, analogous to those established in other fields of materials science, to ensure that the relationships derived from these data remain transferable across materials, water matrices, and system configurations.

## Path toward More Rigorous, Comparable, and Decision-Relevant Sorption Science

Advancing sorption science for TrOC removal requires a coordinated shift toward approaches that prioritize predictive utility, transferability, technical scale-up, and relevance to real-world decision-making. The three areas examined in this review, namely the thermodynamic principles governing sorption equilibria, the design and interpretation of kinetic data, and the evaluation and scale-up of novel sorbents, share a common limitation: prevailing practices generate data that are tied to specific experimental configurations and cannot be readily used to inform process design or material selection or design. Addressing this limitation requires not only improvements in individual experimental practices but also more deliberate alignment among experimental design, modeling frameworks, and performance metrics so sorption data can flow more directly into engineering decisions. Table 4 summarizes key pitfalls and corresponding best-practice recommendations. Future studies should focus on generating sorption data under conditions that enable meaningful comparison across sorbents, contaminants, and treatment scenarios, while preserving the mechanistic insight needed for predictive modeling and material innovation. Therefore, experimental designs are needed that better capture competitive sorption behavior, environmentally relevant concentration regimes, realistic water matrices, and the mass-transfer limitations that ultimately govern treatment performance across scales. For novel sorbents, greater emphasis is also needed on whether performance advantages observed during early-stage laboratory evaluations hold across changes in particle size, material morphology, and process configuration, and whether these advantages remain meaningful when benchmarked against state-of-the-art sorbents under application-relevant conditions. Community-level efforts to standardize experimental protocols, reporting metrics, and benchmarking practices would substantially improve the comparability of sorption data across studies and accelerate the translation of laboratory findings into engineered solutions. The resulting larger, more consistent data sets may also enable machine learning to identify relationships among sorbent properties, water chemistry, operating conditions, and treatment performance, supporting predictive modeling and sorbent design. Collectively, these efforts will enable sorption research to move beyond demonstration of removal toward a more rigorous, comparable, and application-driven science that supports the design and implementation of effective TrOC treatment.

**Table 4:** Best Practices for Designing Experiments to Ascertain and Report Sorption Parameters

Pitfall	Recommendation	Reasoning
Demonstrating high % removal using high sorbent doses and/or TrOC concentrations in ultrapure water.	Test under realistic conditions: Use practical sorbent doses (adjust to achieve 40–60% removal), environmentally relevant TrOC concentrations, and representative water matrices. Report solid-to-liquid ratio.	High sorbent doses and/or TrOC concentrations measured in ultrapure water mask differences among sorbents and generate equilibrium or kinetic parameters that are not representative of application scenarios.
Ignoring competition from DOM, cocontaminants, and/or inorganic ions.	Account for competition: Explicitly evaluate the effects of DOM, cocontaminants, and/or inorganic ions along with bulk parameters such as pH and ionic strength on sorption. Background ions and pH are especially important for ionic/ionizable compounds.	Environmentally relevant water matrices contain constituents that may compete for sorption sites or physically block access to the sorbent pore structure. Bulk water chemistry, such as pH, can change sorbate speciation and sorbent surface charge, thereby affecting sorption.
Relying on the assumption of sorption equilibrium to model breakthrough curves.	Intraparticle and/or boundary layer diffusion kinetics play a major role in solute elution behavior. For the same reason, it is unwise to rely on miscible displacement tests to obtain sorption parameters.	The rates of solute sorption/desorption are seldom instantaneous for sorbents of the types used in remediation, and never for carbonaceous sorbents.
Crushing sorbents to particle sizes well below their intended application size to enable batch testing or to accelerate sorption equilibrium.	Use representative particle sizes: Conduct benchmarking experiments using particle sizes representative of the intended application and report particle size distribution.	Crushing sorbents modifies intraparticle diffusion length scales and may alter pore accessibility and particle structure, resulting in experimental data that are not representative of the sorbent at application scale.
Comparing sorption kinetics using experimental data for sorbents with different particle sizes.	Use sorbents with the same particle size.	For diffusion controlled-processes, sorption rate is inversely proportional to the square of the sorbent particle diameter. For example, when decreasing the sorbent particle size from 500 um to 50 um, the sorption rate would increase by a factor of 100 for a constant intraparticle diffusion coefficient.
Excluding benchmark experiments with conventional sorbents such as activated carbon or ion exchange resins.	Include conventional sorbents in experimental design: Evaluate the performance of activated carbon and/or ion exchange resins in parallel under identical physical (e.g., particle size) and experimental conditions.	Demonstrating removal of a target TrOC alone is insufficient to establish the value of a novel sorbent; performance should be evaluated relative to existing sorption technologies under comparable conditions to determine whether the new material offers a meaningful advancement in sorption affinity, selectivity, kinetics, or operational robustness relative to conventional sorbents used in practice.
Using empirical equilibrium or kinetic parameters tied to a specific batch experiment.	Generate mechanistically meaningful parameters: Use appropriate equilibrium and mass-transfer models to obtain parameters that can predict performance beyond a specific batch experiment. Avoid empirical kinetic models that do not explicitly include mechanistic representation of rate-determining processes. Acknowledge that parameters remain relevant only for the specific water matrix tested.	Empirical parameters are experiment- and matrix-dependent. Mechanistically grounded parameters are essential for interpreting governing processes and predicting performance beyond the conditions tested.
Using conventional batch kinetic experiments to represent granular sorbent behavior in column reactors.	Use flow-through experiments to obtain column-relevant kinetics: Use RSSCTs, short-bed adsorbers, or other types of flow-through experiments to evaluate sorption kinetics for granular sorbents.	Breakthrough data from flow-through experiments can be used to extract mass-transfer coefficients that identify rate-determining processes.
Evaluating promising sorbents without long-term or process-relevant validation.	Validate under realistic operating conditions: Benchmark against state-of-the-art sorbents and use long-term laboratory, pilot-scale, and/or field-relevant testing to assess fouling, biological effects, and mechanical and functional stability.	Eventual technical scale-up requires sustained sorption as well as mechanical and functional stability over the expected operating life of the process.
Assuming sorbent performance and kinetic parameters transfer directly across particle sizes and/or material forms.	Validate scaling across particle sizes and/or material forms: Test particle sizes and material forms that are relevant to deployment to verify that scaling assumptions hold.	Assumptions of constant or proportional diffusivity have been verified for activated carbon and ion exchange resins. Appropriate scaling relationships for other sorbents require careful verification.
Adoption of disadvantageous practices in isotherm construction and fitting.	Follow the 20%-80% rule on solute depletion limits. Eliminate or correct for bottle losses (degradation, evaporation, sorption to reactor). Use appropriate kinetic technique to set equilibration time. Collect sufficient data and treat each datum as an independent experiment. Incorporate measurement error in *C*_w_ into nonlinear regression. Avoid mechanistic overinterpretation of fits.	Working outside solute depletion limits risks large errors in *C*_w_ and/or *q*. Bottle losses risk large error in *q*. When *q* is calculated, “replicates” do not represent true replication. Conventional nonlinear regression assumes no error in *C*_w_, which is not generally true. A best model fit can only support, not assign mechanism.
